# High-Fructose Diet and Chronic Unpredictable Stress Modify Each Other’s Neurobehavioral Effects in Female Rats

**DOI:** 10.3390/ijms252111721

**Published:** 2024-10-31

**Authors:** Sanja Kovačević, Željko Pavković, Jelena Brkljačić, Ivana Elaković, Danijela Vojnović Milutinović, Ana Djordjevic, Vesna Pešić

**Affiliations:** 1 Department of Biochemistry, Institute for Biological Research “Siniša Stanković”—National Institute of Republic of Serbia, University of Belgrade, 142 Despot Stefan Blvd., 11060 Belgrade, Serbia; sanja.kovacevic@ibiss.bg.ac.rs (S.K.); brkljacic@ibiss.bg.ac.rs (J.B.); ivanae@ibiss.bg.ac.rs (I.E.); dvojnovic@ibiss.bg.ac.rs (D.V.M.); djordjevica@ibiss.bg.ac.rs (A.D.); 2 Laboratory of Molecular Neurobiology and Behavior, Department of Neurobiology, Institute for Biological Research “Siniša Stanković”—National Institute of Republic of Serbia, University of Belgrade, 142 Despot Stefan Blvd, 11060 Belgrade, Serbia; zeljko.pavkovic@ibiss.bg.ac.rs

**Keywords:** hyperactivity, thigmotaxis, synaptic plasticity, neuronal activation, corticosterone, CaMKII, glucocorticoid receptor

## Abstract

A pervasive exposure to stressors and the consumption of fructose-containing beverages usually go hand-in-hand in everyday life. In contrast to their metabolic outcomes, their impact on the brain and behavior is still understudied. We examined the behavioral response to a novelty (open field test), the expression of biochemical indicators of neuronal activity (Egr1 and FosB/ΔFosB), the synaptic potentiation (CaMKIIα and pCaMKII^Thr286^), the synaptic plasticity (synaptophysin, PSD95, gephyrin, and drebrin), and the GABAergic system (parvalbumin and GAD67), along with the glucocorticoid receptor (GR) and AMPK, in the medial prefrontal cortex of female Wistar rats subjected to liquid fructose supplementation (F), chronic unpredictable stress (S), or both (SF) over 9 weeks. The only hallmark of the F group was an increased expression of pCaMKII^Thr286^, which was also observed in the S group, but not in the SF group. The SF group did not show hyperactivity, a decreased expression of FosB, or an increased expression of parvalbumin, as the S group did. The SF group, as with the S group, showed a decreased expression of the GR, although the basal level of corticosterone was unchanged. The SF group showed, as de novo marks, thigmotactic behavior, increased drebrin, and decreased gephyrin expression. These findings suggest that the long-term consumption of fructose, which itself has subtle neurobehavioral consequences, in combination with stress prevents some of its effects, but also contributes to novel outcomes not seen in single treatments.

## 1. Introduction

Fructose is a monosaccharide that is naturally present in fruit, vegetables, and honey. When ingested for a prolonged time as an additive to meals, it might produce metabolic syndrome and obesity [1,2]. However, the impact of fructose on human health is still a source of controversy (emphasized in [3]), with the energy control, food source, feeding patterns, and levels at which fructose is normally consumed as crucial to gaining a proper understanding of the health outcomes [4,5]. Fructose consumption has increased dramatically worldwide in recent decades [6], but it can hardly be separated from the ubiquitous presence of unpredictable stressful situations. Moreover, there are indications that millennials (ages 18–33 years) as a generational group have the highest levels of stress of any other age group and that women experience higher levels of stress compared with men [7,8,9]. Therefore, considering the outcome of chronic fructose intake per se and in combination with chronic unpredictable stress (CUS) in female rodents particularly increases the external validity of the data obtained in preclinical research.

The impact of fructose on the brain has been less explored than its impact on other organs, and published studies, which have been largely performed on male rodents, mainly emphasize metabolic and electrophysiological changes in the hippocampus, thalamus, and hypothalamus [10], accentuating cognitive impairment as a consequence [11]. The medial prefrontal cortex (mPFC), which, in both humans and rodents, represents a key region for high executive functions, decision making, and mood control [12,13], has not been examined in preclinical research dealing with fructose intake. This is surprising, since there are indications that fructose reduces the activation of the mPFC and promotes the disinhibition of feeding/the consumption of palatable foods in humans [14]. The PFC is extensively implicated in behavioral disinhibition, a temperamental tendency characterized by a novelty- and sensation-seeking personality and the negligence of potential or actual dangers in humans [15]. Recently, the hypothesis has been presented that fructose metabolism represents a common evolutionary pathway of survival and that a high intake of fructose leads to a hyperactive foraging response, thus linking sugar intake with behavioral disorders associated with impulsivity and hyperactivity, whose occurrence has increased in parallel with the obesity epidemic [16]. Yet, there is still no experimental evidence on how this is modeled in preclinical studies, i.e., whether findings in laboratory rodents confirm that a chronic/continuous fructose intake impacts behavioral control, food intake, and activity-regulated processes in the mPFC and how these are modeled in the presence of unpredictable stressful situations. This is an important aspect when considering the adverse effects of fructose intake, since the widely accepted view is that sugar has stress-dampening effects, due to which chronic stress promotes sugar intake [17]. However, these studies have actually addressed the stress-relieving properties of sucrose (a disaccharide composed of glucose and fructose; herbivores and omnivores are highly adapted to use sucrose for energetic and biosynthetic needs [18]), while the stress-relieving properties of fructose as the isolated monosaccharide still need clarification.

In the present study, we aimed to examine the psychoneuroendocrine outcome of chronic (9-week) fructose consumption and CUS per se, as well as their combination, in female rats. We considered CUS-related biochemical autographs that are already related to and are considered mechanistic biomarkers of the mPFC hypofunction. Specifically, the decreased expression of the glucocorticoid receptor (GR) was reported as a mark of a disrupted glucocorticoid negative feedback system overall, leading to limited influence of the PFC on the control of stress reactivity and behavior [19,20]. CUS exposure significantly decreased the expression of GABA synthetic enzyme glutamic acid decarboxylase 67 (GAD67) [21]. It also decreased the level of the scaffolding proteins gephyrin (GABA-A receptor-associated postsynaptic protein) and postsynaptic density protein 95 (PSD-95; enriched at glutamatergic synapses) in the PFC [21,22,23]. The loss of spines and dendrites in the mPFC neurons due to stress has been reported [24,25]. Data on the calcium/calmodulin-dependent kinase II alpha (CaMKIIα) phosphorylated at Thr^286^ (pCaMKII^Thr286^), which is almost exclusive of synapses receiving glutamatergic terminals and has an important role in synaptic potentiation [26,27], are lacking. Novel findings have also highlighted that CUS increases parvalbumin (PV) expression and the number of PV+ neurons (fast-spiking GABAergic neurons that are powerful regulators of local network activities), particularly in female mice, along with heightened anxiety-like behaviors [28,29]. Moreover, one of the well-known autographs of the CUS is the increased expression of delta FosB (ΔFosB) in the PFC [30]. It is also known that stress affects the energy metabolism of neurons and that the CUS-induced inactivation of AMP-activated protein kinase (AMPK, a crucial cellular energy sensor) in the cortex might be a mechanism by which CUS causes anxiety/depression-like behaviors [31,32].

We hypothesized that the behavioral and biochemical outcomes of liquid fructose intake would be related to the presence of CUS, i.e., that the outcomes of CUS would change due to fructose intake. To address this hypothesis, we examined the following: the consummatory behavior towards regular food and liquid intake of the animals during the exposure period; the behavioral response of the animals to an inescapable novel environment after the exposure period; biochemical adaptations in the mPFC after the exposure period, addressing the expression of biochemical indicators of neuronal activity (Egr1 and FosB/ΔFosB) and synaptic potentiation (CaMKIIα and pCaMKII^Thr286^), protein markers of synaptic plasticity (synaptophysin, PSD95, gephyrin, and drebrin), protein markers of the GABAergic system (parvalbumin and GAD67), and AMPK as a cellular energy sensor protein; the level of corticosterone, as a classic endocrine response to stress, during and after the exposure period; and its receptor (GR) in the mPFC.

The findings of this study, conducted using a physiologically relevant fructose concentration (10%, [33]) and CUS as a non-habituation stress regimen [34], together with well-characterized metabolic outcomes of the same paradigm [35,36,37,38], contribute to the characterization of an important preclinical model of the effects of fructose and stress on health in females.

## 2. Results

### 2.1. Feeding Behavior in Stress-Exposed, Fructose-Fed, and Stress-Exposed Fructose-Fed Female Rats

**Figure 1:**
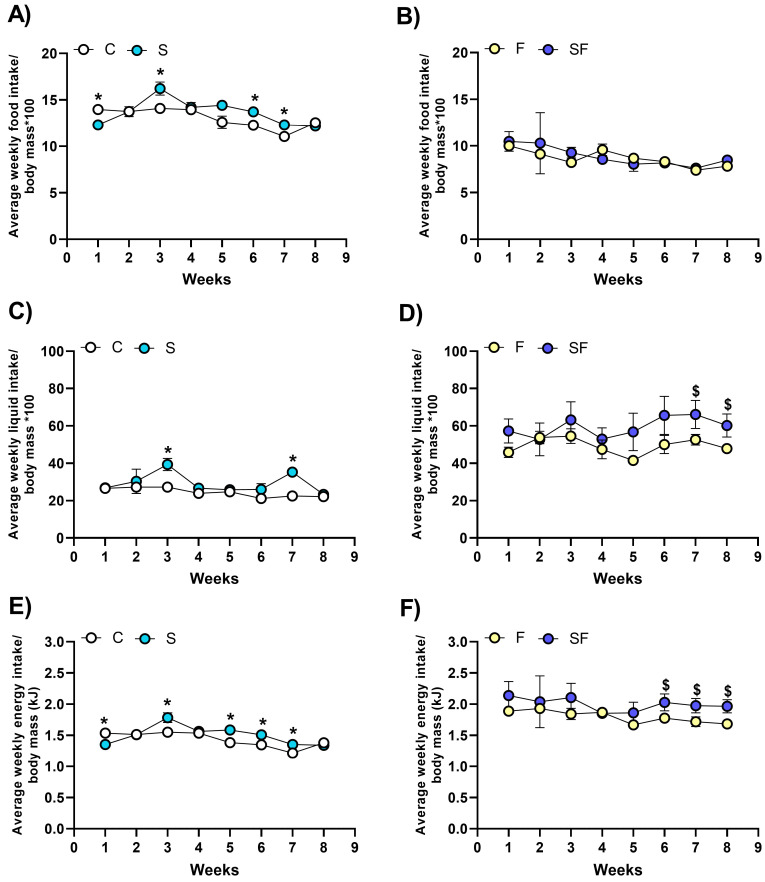
Feeding behavior in stress-exposed, fructose-fed, and stress-exposed fructose-fed female rats. The food, fluid, and energy intake were monitored throughout the course of the experiment and are expressed as the average weekly intake per body mass of the animals. Comparisons were made between the control group (C) and the stress-exposed group (S) to assess the influence of the stress protocol on the intake of water, standard chow, and energy (panels (**A**,**C**,**E**), respectively), and between the fructose-fed (F) and stress-exposed fructose-fed (SF) group to assess the influence of stress on the intake of the fructose solution, standard chow, and energy (panels (**B**,**D**,**F**), respectively). Comparisons between the groups that had the fructose solution as their only source of fluid with the groups that had water as the only source of fluid were intentionally avoided regarding the mentioned parameters. * *p* < 0.05 vs. control group, ^$^ *p* < 0.05 vs. fructose-fed group.

The food, fluid, and energy intake of female rats was monitored throughout the course of the experiment (Figure 1). The values measured in week 9 were excluded from the calculations, as the animals were subjected to behavioral testing and their food and liquid intake was disturbed. Comparisons were made between the control group and the CUS-exposed group to assess the influence of the stress protocol on the intake of water and standard chow, and between the fructose-fed and CUS-exposed fructose-fed group to assess whether and how the fructose and standard chow intake changed in the presence of CUS.

Compared to the control, the CUS-exposed rats had an increased food intake in weeks 1, 3, 6, and 7 (Figure 1A, *p* < 0.05), an increased water intake in weeks 3 and 7 (Figure 1C, *p* < 0.05), and an increased energy intake in weeks 1, 3, 5, 6, and 7 (Figure 1E, *p* < 0.05).

Compared to the group of fructose-fed rats, the CUS-exposed fructose-fed rats did not differ regarding their food intake (Figure 1B), but their intake of fructose solution and their total energy intake were increased during the last weeks of the experiment (fructose intake in weeks 7 and 8, Figure 1D, *p* < 0.05; total energy intake in weeks 6, 7, and 8, Figure 1F, *p* < 0.05).

### 2.2. Behavioral Response of Stress-Exposed, Fructose-Fed, and Stress-Exposed Fructose-Fed Female Rats to an Inescapable Novel Environment

**Figure 2:**
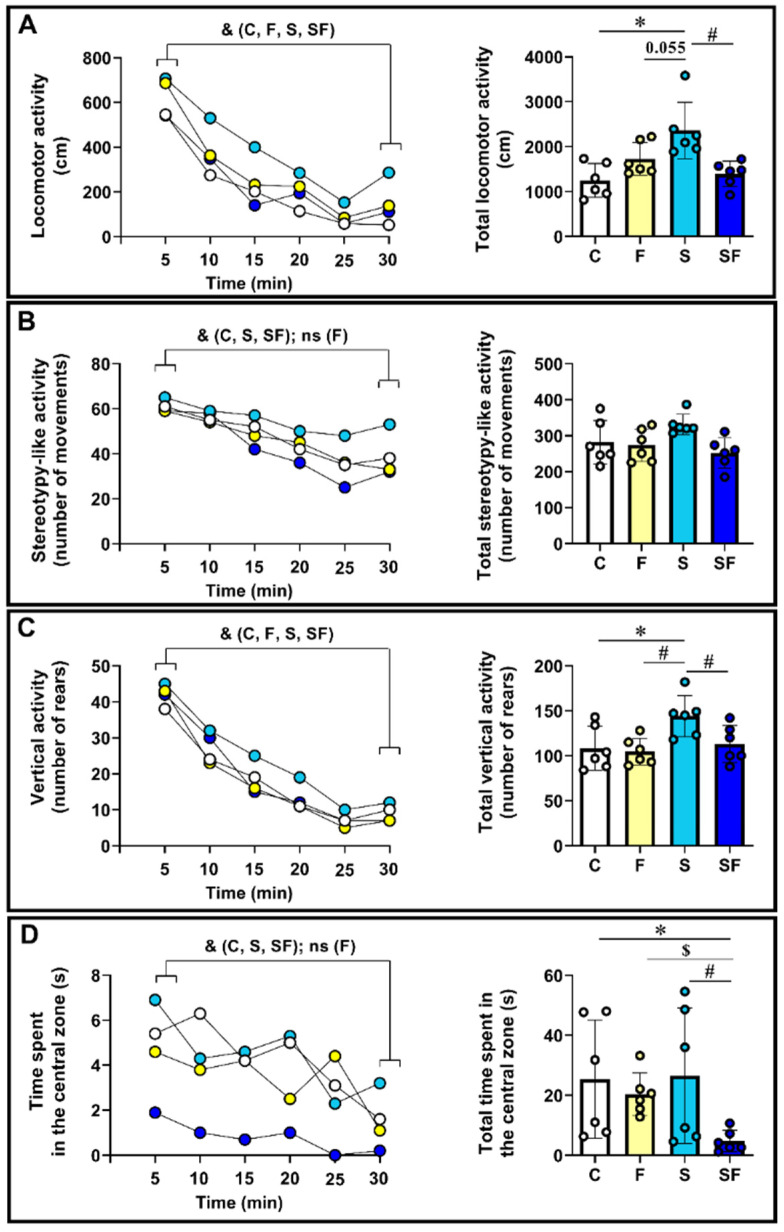
Behavioral response of stress-exposed, fructose-fed, and stress-exposed fructose-fed female rats to an inescapable novel environment. The line graphs (left side of panels (**A**–**D**)) illustrate the dynamics of changes in the examined parameters over time and represent the mean value of the group. For the total activity (right side of panels (**A**–**D**)), the data are presented as the mean ± SD, with individual data plots along the column bars. ^&^ *p* < 0.05, Wilcoxon test; * *p* < 0.05 vs. control group, U-test; ^$^ *p* < 0.05 vs. fructose-fed group, U-test; ^#^ *p* < 0.05 vs. stress-exposed group, U-test. C—control, F—fructose-fed, S—stress-exposed, SF—stress-exposed fructose-fed.

Locomotor activity: All the experimental groups of animals showed within-session habituation (Figure 2A, left panel, ^&^ *p* < 0.05, Wilcoxon test; the activity scored during the first compared to the activity scored during the last five minutes). The Kruskal–Wallis *H* test revealed a significant influence of the treatments on the locomotor activity count (Figure 2A, right panel; H(3) = 12.77, *p* = 0.005). The between-group comparison showed that the locomotor activity count was the highest in stress-exposed rats, i.e., it was significantly higher in this group compared to the control (* *p* < 0.05) and the stress-exposed fructose-fed group (^#^ *p* < 0.05), while the comparison with the fructose-fed group showed a marginally significant *p*-value (*p* = 0.055).

Stereotypy-like activity: The control rats, as well as the stressed and stress-exposed fructose-fed rats, showed within-session habituation (Figure 2B, left panel, ^&^ *p* < 0.05, Wilcoxon test). In the fructose-fed rats, the analysis of this parameter showed marginal significance (Figure 2B, left panel *p* = 0.074, Wilcoxon test). The Kruskal–Wallis *H* test revealed a non-significant influence of the treatments on the stereotypy-like activity count (Figure 2B, right panel; H(3) = 7.033, *p* = 0.071).

Vertical activity: All the experimental groups of animals showed within-session habituation (Figure 2C, left panel, ^&^ *p* < 0.05, Wilcoxon test). The Kruskal–Wallis *H* test revealed a significant influence of the treatments on the vertical activity count (Figure 2C, right panel; H(3) = 8.649, *p* = 0.034). This parameter was the highest in the stress-exposed rats, i.e., it was significantly higher in this group compared to the control (* *p* < 0.05), fructose-fed (^$^ *p* < 0.05), and stress-exposed fructose-fed (^#^ *p* < 0.05) rats.

Time spent in the central zone: The control rats, as well as the stress-exposed and stress-exposed fructose-fed rats, showed within-session habituation (Figure 2D, left panel, ^&^ *p* < 0.05, Wilcoxon test). In the fructose-fed rats, the analysis of this parameter showed marginal significance (Figure 2D, left panel, *p* = 0.080, Wilcoxon test). The Kruskal–Wallis *H* test revealed a significant influence of the treatments on the total time spent in the central zone (Figure 2D, right panel; H(3) = 9.479, *p* = 0.024). The between-group comparisons revealed that this parameter was significantly less in the group of stress-exposed fructose-fed rats compared to the control (* *p* < 0.05), fructose-fed (^$^ *p* < 0.05), and stress-exposed (^#^ *p* < 0.05) rats.

### 2.3. The Expression of Protein Indicators of Neuronal Activation and Synaptic Potentiation in the Medial Prefrontal Cortex of Stress-Exposed, Fructose-Fed, and Stress-Exposed Fructose-Fed Female Rats

**Figure 3:**
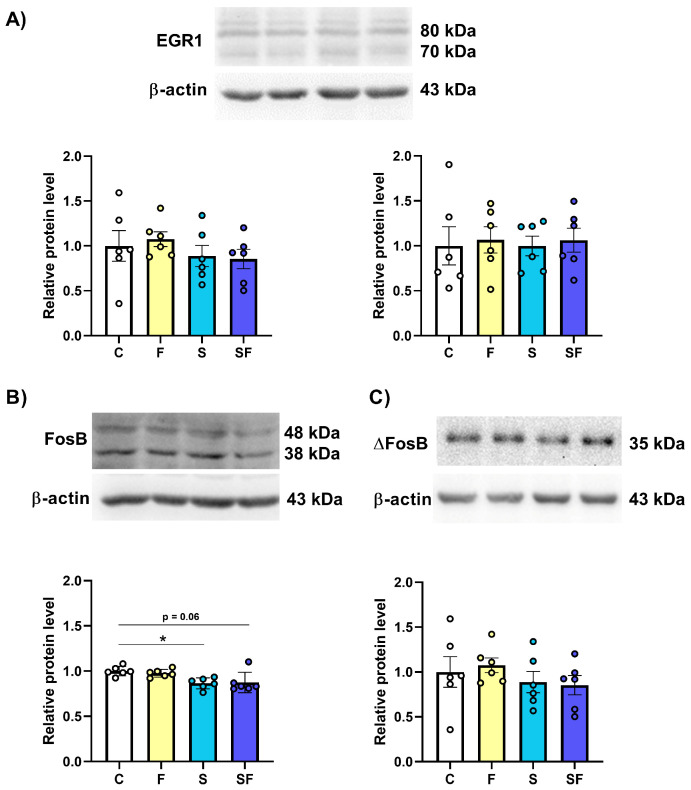
The expression of protein indicators of neuronal activation in the medial prefrontal cortex of stress-exposed, fructose-fed, and stress-exposed fructose-fed female rats. The data are presented graphically, as the mean ± SD, with individual data plots along the column bars. Each graph is provided with representative immunoblots of (**A**) the EGR1 80 kDa isoform—left panel; the EGR1 70 kDa isoform—right panel; (**B**) FosB; (**C**) ΔFosB; and β-actin, which was used as a loading control. * *p* < 0.05 vs. control group. C—control, F—fructose-fed, S—stress-exposed, SF—stress-exposed fructose-fed.

EGR1, FosB, and ΔFosB were analyzed in the mPFC as indirect markers to measure neuronal activation, with ΔFosB being uniquely stable among the examined proteins. The treatments did not influence the expression of EGR1 or ΔFosB (Figure 3A,B,D), but influenced the FosB expression (Figure 3C, Kruskal–Wallis *H* test: H(3) = 11.39; *p* < 0.01). Compared to the control group, the stress-exposed rats on a standard diet showed a lower FosB protein level (Figure 3C, * *p* < 0.05), while the stress-exposed fructose-fed group showed a marginally significant decrease (*p* = 0.06).

**Figure 4:**
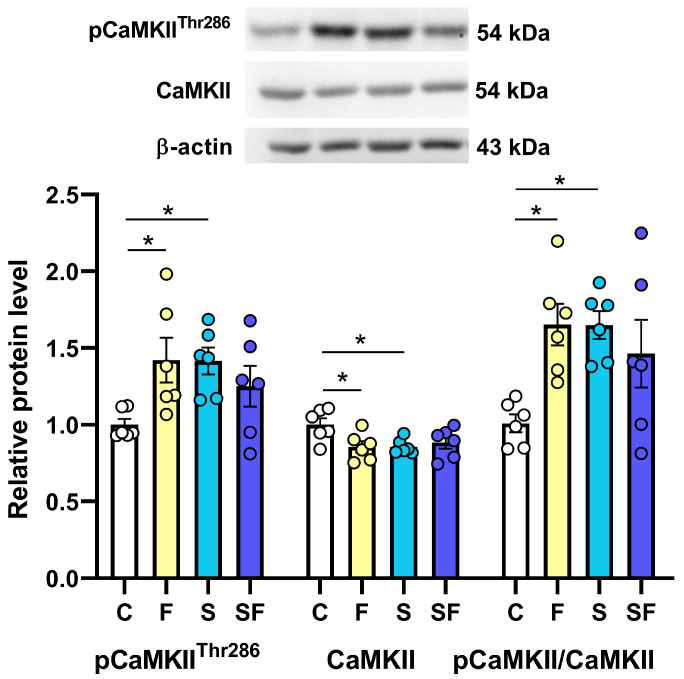
The expression of CaMKIIα and its phosphorylated form, pCaMKII^Thr286^, a biochemical indicator of synaptic potentiation, in the medial prefrontal cortex of stress-exposed, fructose-fed, and stress-exposed fructose-fed female rats. The data are presented graphically, as the mean ± SD, with individual data plots along the column bars. The graph is provided with representative immunoblots of the proteins of interest and β-actin, which was used as a loading control. * *p* < 0.05 vs. control group. C—control, F—fructose-fed, S—stress-exposed, SF—stress-exposed fructose-fed.

CaMKIIα is the most abundant protein in excitatory synapses and is central to synaptic plasticity. A brief Ca^2+^ stimulus in the synapse can lead to Thr286 autophosphorylation, with CaMKII phosphorylation at Thr286 (pCaMKII^Thr286^) representing an essential event in the induction of long-term potentiation. The statistical analysis revealed that the applied treatments significantly influenced the expression of pCaMKII^Thr286^ (Figure 4; H(3) = 9.55; *p* < 0.05), the total CaMKII (Figure 4; F(3, 20) = 3.65; *p* < 0.05), and the ratio between the phosphorylated and total CaMKII (Figure 4; F(3, 20) = 4.65; *p* < 0.05). Compared to the control, the expression of pCaMKII^Thr286^ was increased and the level of total CaMKII was decreased in the groups of fructose-fed and stress-exposed rats, overall resulting in a significantly increased pCaMKII^Thr286^/CaMKII ratio in these groups (* *p* < 0.05).

### 2.4. The Expression of Protein Indicators of Altered Synaptic Plasticity in the Medial Prefrontal Cortex of Stress-Exposed, Fructose-Fed, and Stress-Exposed Fructose-Fed Female Rats

**Figure 5:**
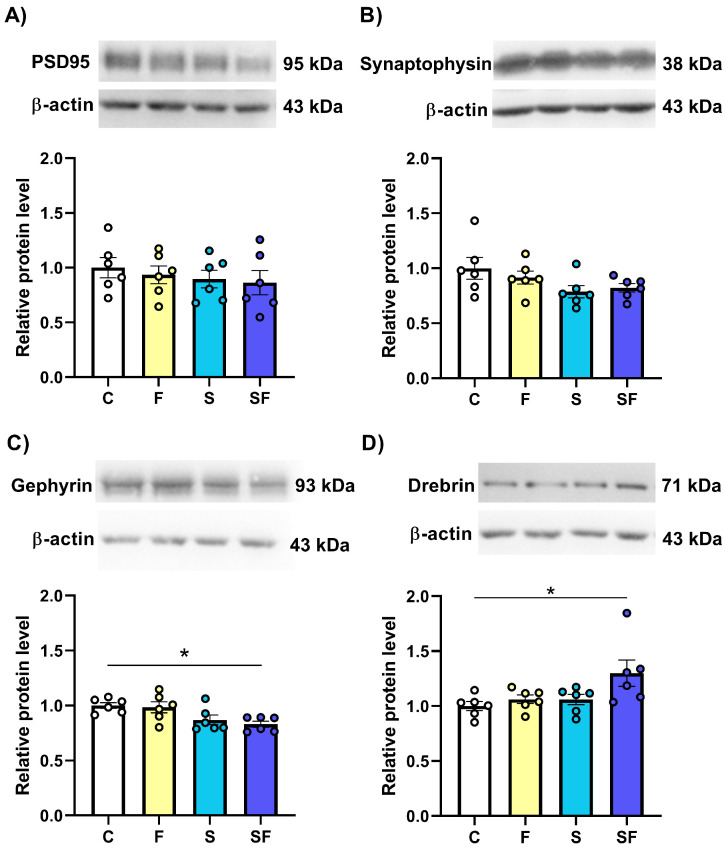
The expression of protein indicators of altered synaptic plasticity in the medial prefrontal cortex of stress-exposed, fructose-fed, and stress-exposed fructose-fed female rats. The data are presented graphically, as the mean ± SD, with individual data plots along the column bars. Each graph is provided with representative immunoblots of (**A**) PSD95, (**B**) synaptophysin, (**C**) gephyrin, (**D**) drebrin, and β-actin, which was used as a loading control. * *p* < 0.05 vs. control group. C—control, F—fructose-fed, S—stress-exposed, SF—stress-exposed fructose-fed.

Among the different protein markers of synaptic plasticity, the current study analyzed the expression of synaptophysin, PSD95, gephyrin, and drebrin. The expression level of PSD95, the major scaffolding protein in the postsynaptic density of excitatory synapses, remained unchanged in the mPFC of fructose-fed, stress-exposed, and fructose-fed stress-exposed rats compared to the control group (Figure 5A). The same was observed regarding the expression of synaptophysin, an integral membrane glycoprotein of presynaptic vesicles, which is widely used as a presynaptic marker (Figure 5B). However, the Kruskal–Wallis *H* test revealed significant differences in the expression of gephyrin, the most extensively studied scaffold protein responsible for organizing the inhibitory postsynaptic density (Figure 5C; H(3) = 11.23, *p* < 0.05), and drebrin, the protein that is abundant within dendritic spines at postsynaptic excitatory synapses (Figure 5D; H(3) = 7.48, *p* < 0.05). The between-group comparisons revealed that, compared to the control group, the mPFC of stress-exposed fructose-fed rats had a significantly decreased expression of gephyrin (Figure 5C, * *p* < 0.05) and a significantly increased expression of drebrin (Figure 5D, * *p* < 0.05).

### 2.5. The Expression of the Glucocorticoid Receptor in the Medial Prefrontal Cortex and the Level of Corticosterone in the Blood of Stress-Exposed, Fructose-Fed, and Stress-Exposed Fructose-Fed Female Rats

**Figure 6:**
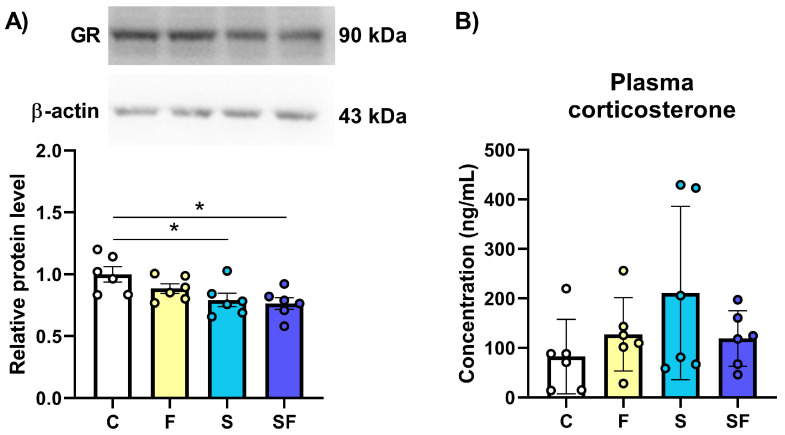
The expression of the glucocorticoid receptor (**A**) in the medial prefrontal cortex and the level of corticosterone in the blood (**B**) of stress-exposed, fructose-fed, and stress-exposed fructose-fed female rats. The data are presented graphically, as the mean ± SD, with individual data plots along the column bars. A graph representing changes in the GR expression is given with representative immunoblots of the protein of interest and β-actin, which was used as a loading control. * *p* < 0.05 vs. control group. C—control, F—fructose-fed, S—stress-exposed, SF—stress-exposed fructose-fed.

The expression of the GR in the mPFC of female rats was significantly influenced by the treatments (Figure 6A; F(3, 20) = 4.27, *p* < 0.05). Compared to the control, the level of the GR protein was significantly decreased in the mPFC of stress-exposed rats, regardless of their diet (* *p* < 0.05). The plasma corticosterone level at the end of the exposure period was not significantly different between the groups (Figure 6B).

### 2.6. The Efficacy of a 9-Week CUS Protocol in Achieving an Increase in Circulating Corticosterone Levels in Female Rats

**Figure 7:**
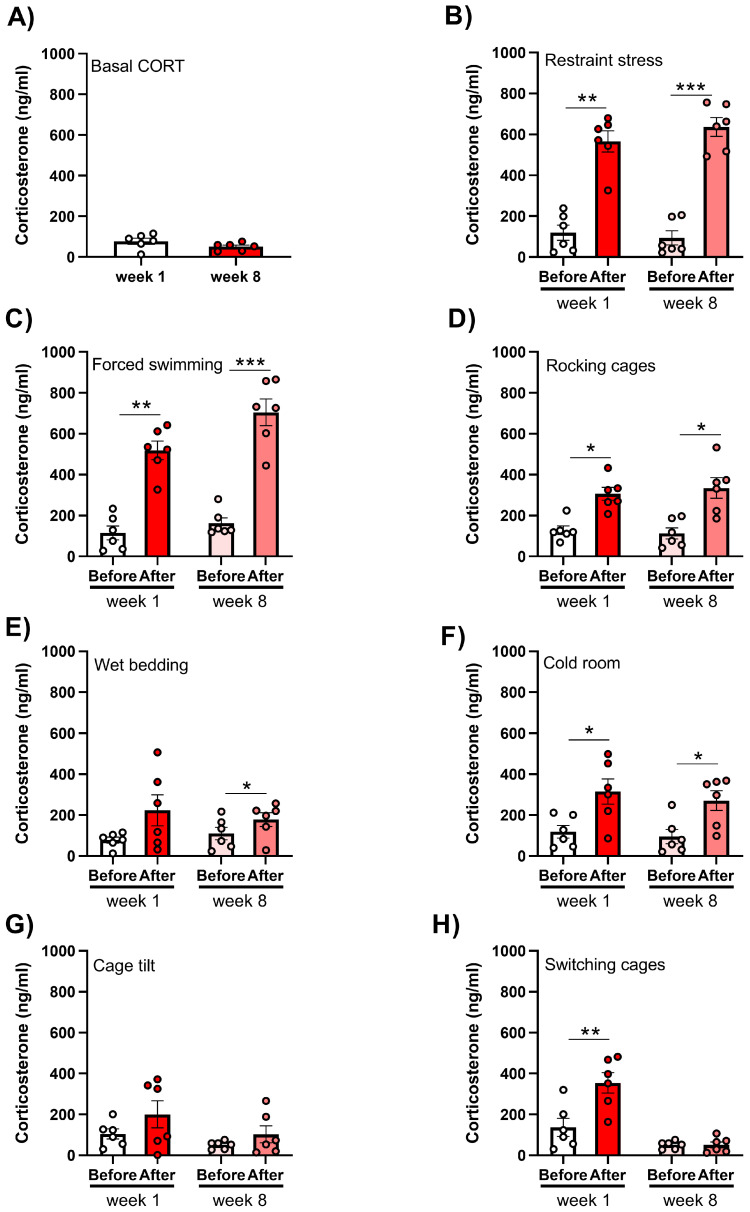
The efficacy of a 9-week chronic unpredictable stress protocol in achieving an increase in the circulating corticosterone levels in female rats. To assess (**A**) the basal corticosterone as well as the corticosterone level before and immediately after the application of (**B**) restraint stress, (**C**) forced swimming, (**D**) rocking cages, (**E**) wet bedding, (**F**) a cold room, (**G**) cage tilt, and (**H**) cage switching, blood was collected via a tail snip at experimental weeks 1 and 8. The data are presented graphically, as the mean ± SD, with individual data plots along the column bars. * *p* < 0.05, ** *p* < 0.01, *** *p* < 0.001, paired *t*-test.

To assess whether and to which stressors the exposed experimental animals became accustomed, their plasma corticosterone was measured at week 1 and week 8. The basal level of corticosterone, defined as (nadir) morning corticosterone, was not significantly different at week 8 compared to week 1 (Figure 7).

A statistical analysis (paired *t*-test) revealed that, of all the applied stressors (forced swimming, wet bedding, cage tilting, restraint stress, rocking cages, cold room, and switching cages), only wet bedding, cage tilting, and switching cages failed to produce a consistent increase in the plasma corticosterone level (Figure 7; switching cages only at week 1, ** *p* < 0.01; wet bedding only at week 8, * *p* < 0.05). For the other stressors, a significant increase and a comparable corticosterone rise were detected at both week 1 and week 8 (Figure 7; restraint: week 1, ** *p* < 0.01, week 8, *** *p* < 0.001; forced swimming: week 1, ** *p* < 0.01, week 8, *** *p* < 0.001; rocking cages: week 1, * *p* < 0.05, week 8, * *p* < 0.05; cold room: week 1, * *p* < 0.05, week 8, * *p* < 0.05).

These results confirmed the efficacy of a 9-week CUS protocol in achieving an increase in corticosterone levels after the application of specific stressors.

### 2.7. The Expression of Protein Markers of the GABAergic System in the Medial Prefrontal Cortex of Stress-Exposed, Fructose-Fed, and Stress-Exposed Fructose-Fed Female Rats

**Figure 8:**
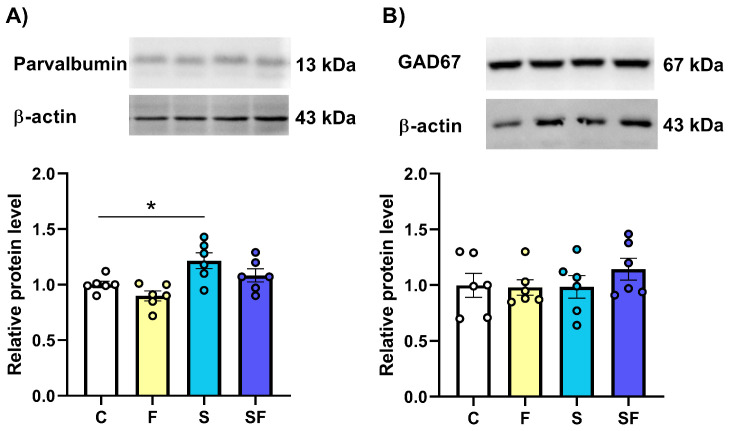
The expression of parvalbumin (**A**) and GAD67 (**B**) in the medial prefrontal cortex of stress-exposed, fructose-fed, and stress-exposed fructose-fed female rats. The data are presented graphically, as the mean ± SD, with individual data plots along the column bars. Each graph is given with representative immunoblots of the protein of interest and β-actin, which was used as a loading control. * *p* < 0.05 vs. control group. C—control, F—fructose-fed, S—stress-exposed, SF—stress-exposed fructose-fed.

The expression of parvalbumin, a calcium-binding protein that is important for the modulation of intracellular calcium dynamics in neurons, was significantly influenced by the treatments, as indicated by a one-way ANOVA (F(3, 20) = 6.35, *p* < 0.01). The between-group comparisons showed that, compared to the control group, the parvalbumin protein level was significantly higher only in the mPFC of stress-exposed rats on a standard diet (Figure 8A, * *p* < 0.05). The level of GAD67, an enzyme that metabolizes glutamate to GABA, was not significantly different between groups (Figure 8B).

### 2.8. The Expression of AMPK in the Medial Prefrontal Cortex of Stress-Exposed, Fructose-Fed, and Stress-Exposed Fructose-Fed Female Rats

**Figure 9:**
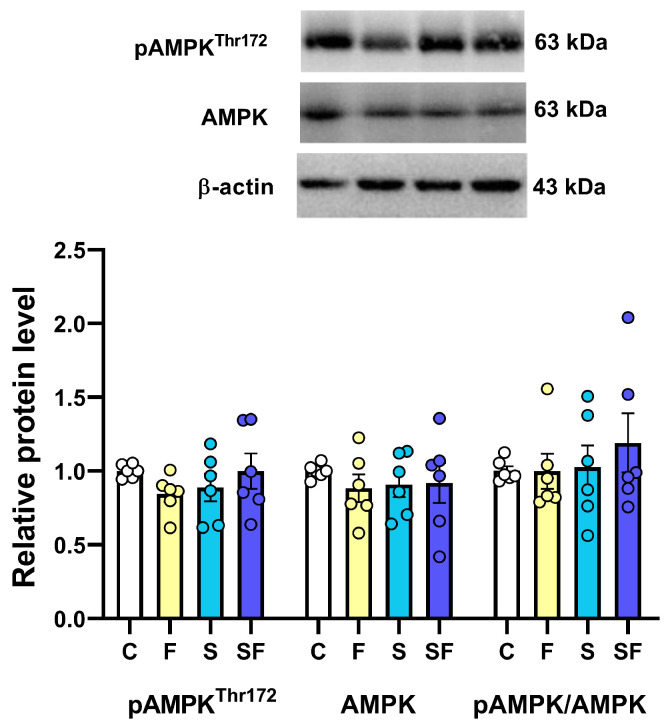
The expression of AMPK, a cellular energy sensor protein, in the medial prefrontal cortex of stress-exposed, fructose-fed, and stress-exposed fructose-fed female rats. The data are presented graphically, as the mean ± SD, with individual data plots along the column bars. A graph is given with representative immunoblots of the proteins of interest and β-actin, which was used as a loading control. C—control, F—fructose-fed, S—stress-exposed, SF—stress-exposed fructose-fed.

There was no significant influence of the treatments on the level of total AMPKα; AMPKα phosphorylated at Thr172 (pAMPK^Thr172^), a phosphorylation that plays a crucial role in regulating the catalytic activity and overall functionality of AMPK; or the pAMPK^Thr172^/AMPK ratio in the mPFC of the examined animals (Figure 9).

The summary of the obtained results is given in the Table 1.

## 3. Discussion

Understanding the impact of the long-term consumption of fructose, as an isolated sugar, and stress on health is still limited. This research contributes by showing, in a rodent model, that the long-term consumption of fructose, which itself has subtle neurobehavioral consequences, in combination with stress prevents some of its effects, but also promotes novel outcomes not seen in single treatments.

Fructose consumption was potentiated in CUS-exposed female rats after 6 weeks of exposure. Fructose consumption per se did not change the novelty-induced behavioral response of the animals. However, in combination with CUS, fructose consumption was associated with both the calming of a CUS-induced increase in locomotor and rearing responses to the novelty and the de novo induction of extreme thigmotactic behavior (avoidance of the central zone of the arena). In the rat model, a hyperlocomotor response to an inescapable novel arena was found to be the best parameter for monitoring the progression of CUS, with the suggestion that such a behavioral response is a consequence of impaired arousal-inhibition and information-processing systems [39]. In rodents, increased locomotor activity in a novel inescapable environment models sensation-seeking and is used in preclinical studies to examine the role of behavioral/personality traits in the vulnerability to drug use [40,41]. Thigmotaxis is an evolutionarily conserved adaptive response to open spaces that could be related to anxiety disorders [42,43]. Overall, the obtained data suggest that fructose intake mitigates some of the behavioral effects of CUS, which may explain, at least in part, the increase in fructose intake in the stress-exposed fructose-fed group. But this is not a clear benefit, since it is coupled with the promotion of anxiety-like behavior. The observed findings are important, considering that the functional contribution of the stress-relieving properties of fructose as the isolated monosaccharide have still not been clarified in the literature, regardless of the general attitude that sugar has stress-dampening effects [17]. Our findings do not support the view that fructose intake is related to hyperactive behavior [16], at least in females, leaving open the possibility that this relation might be sex-related. This remains to be investigated in future research.

The sugar/sweetener quantity has been shown to be significantly positively correlated with all mental health disorder prevalence values (i.e., anxiety disorders, mood disorders, impulse control disorders, and substance use disorders), with the suspicion that this relationship may not be direct and that an additional variable may contribute simultaneously to a higher sugar consumption and a greater susceptibility to mental illness [44]. Our findings highlight that this additional variable could be CUS, which contains uncontrollability and chronicity as two parameters important for stress–illness relationships [45]. Because of this, CUS has a special value in replicating the human condition in experimental animals [46].

In order to gain a deeper understanding of whether the continuous intake of fructose alone, in the absence of the additional intake of palatable/energy-dense food, can modify the impact of CUS on the mPFC, we performed several biochemical analyses. Our findings indicated that fructose intake does not mitigate a stress-induced decline in the GR expression in the mPFC. However, we could not exclude the possibility that there were changes in the GR expression within the mPFC in the stress-exposed fructose-fed group, as there is a marked functional heterogeneity of glucocorticoid action in the ventral mPFC [19,20]. Next, we observed an increased expression of drebrin, a protein that regulates dendritic spine morphology, size, and density [47], in the stress-exposed fructose-fed female rats, while the level of this protein was unchanged in the fructose- and CUS-exposed groups. An altered dendritic morphology/atrophy of pyramidal neurons of the mPFC is one of the main outcomes of chronic stress [48,49,50], so one could expect a decrease in drebrin expression in the CUS-exposed group. However, it is known (but largely overlooked in generalized conclusions about the effects of stress) that the mPFC is sexually dimorphic and that the effect of stress on the dendritic morphology in the mPFC is sex-dependent [50,51]. The finding that drebrin expression was increased in the stress-exposed fructose-fed female rats can be viewed as a risk for dendritic hypertrophy and excitotoxicity, particularly considering that the expression of gephyrin, which is crucially involved in adjusting the strength of inhibitory transmission in response to changes in network activity [52], was decreased only in the mPFC of this experimental group. It should be noted that our study did not reveal changes in the expression of gephyrin or GAD67, nor in the expression of PSD95 in the mPFC of CUS-exposed female rats, which is generally not in agreement with previous studies addressing the biochemical consequences of CUS [21,22,23]. Again, this discrepancy may have been due to the different sex of the animals and the duration of the stress protocol used in the present and cited studies. This explanation also helps develop an understanding of the absence of changes in ΔFosB expression [30]. Importantly, we observed a decreased expression of the FosB protein in the mPFC of the CUS-exposed rats and a marginally significant decrease in the mPFC of the rats exposed to both CUS and fructose. These findings implicate that a decreased FosB expression in the mPFC may be considered a CUS-related signature in the present experimental paradigm, as a part of a repressive feedback effect related to the chronicity of the treatment [53]. In this sense, we proved that animals exposed to CUS do not become accustomed to the experimental protocol, that is, they react to the applied stressors by increasing the level of circulating corticosterone immediately after the treatment, but have an unchanged basal corticosterone level. The preserved response to CUS was also confirmed by the reduced expression of the GR in the CUS-exposed groups (already discussed above).

Fructose feeding abolished the CUS-associated increase in the parvalbumin expression in the mPFC of female rats. We did not detect changes in AMPK expression/phosphorylation in the mPFC in any of the rat groups tested, implying that none of the treatments affected the cellular energy status or that homeostasis was achieved due to the chronicity of treatment. Increased parvalbumin expression and deregulated AMPK have been considered important in mediating anxiety/depression-like behaviors related to CUS exposure [28,29,32]. Our results suggest that these biochemical correlates were not crucially important for the expression of anxiety-like behavior (increased thigmotaxis) in the fructose-fed group of female rats exposed to stress.

Of all the examined proteins in the mPFC (summary given in Table 1), the increased expression of pCaMKII^Thr286^ (that is, the increased expression of the active form in the total amount of CaMKIIα protein) appeared as the biochemical autograph of long-term fructose feeding in female rats.

Interestingly, the same outcome was observed in the stress-exposed group and was absent in the fructose-fed stress-exposed group. According to the literature, pCaMKII^Thr286^ is the autonomously active form of CaMKII and represents a molecular memory of a brief Ca^2+^ stimulus in the synapse, making CaMKII activity persist even after the Ca^2+^ concentration falls to baseline levels [26,27,54,55]. Thus, an increased expression of pCaMKII^Thr286^ in the mPFC of fructose-fed rats is evidence that long-term fructose intake has a central effect, as it is lost due to the parallel influence of CUS. Given that activated CaMKII is sufficient to initiate the long-term potentiation of synaptic strength and is important in certain forms of learning [27], as well as the fact that an increase in pCaMKII in the mPFC is associated with addictive behavior [56], additional behavioral testing is needed to gain insight into the functional outcomes of this finding.

The prevalence of anxiety disorders, encompassing generalized anxiety disorder, panic disorder, and agoraphobia, is significantly higher in women than in men, and metabolic syndrome significantly increases the risk of anxiety disorders [57,58]. There are suggestions that, in the rat model, chronic fructose consumption for several weeks could serve as a model to explore the influence of peripheral metabolism on brain function and plasticity [59]. However, our previous studies, which focused on the metabolic effects of CUS exposure and fructose feeding in female rats, showed that fructose and CUS exhibited tissue-specific effects without classical signs of metabolic syndrome, since obesity, systemic insulin resistance, and dyslipidemia (high triglycerides and low HDL cholesterol) were not detected in the female rats. Instead, a 9-week-long supplementation with a 10% fructose solution induced tissue-specific metabolic disturbances, for instance, adipogenesis, inflammation, and insulin resistance in the visceral adipose tissue without the development of obesity [38]. The findings of the present study, along with the above discussed facts, implicate that the outcomes of fructose feeding on the brain should not be perceived purely as secondary, which is supported by the facts that, in some brain regions (i.e., the hippocampus), biochemical and structural alterations are evident after a week of fructose consumption (8% and 15% solution), in the absence of metabolic syndrome markers [60].

The lack of neurobehavioral outcomes due to an unlimited fructose intake, CUS exposure, and the combination of these two interventions over time can be considered limitations of the study. Accordingly, the presented and discussed results indicate only the final outcome of long-term treatments, but not the evolution of the neurobehavioral responses over time. This certainly deserves to be examined in future research, especially considering the finding that the duration of the exposure is a significant variable when assessing the impact of CUS on fructose intake. Accordingly, shorter periods of exposure to unlimited fructose intake, CUS, or their combination could have a different neurobehavioral autograph. The lack of a histological analysis of the brain can be considered a limitation as well and should be performed in future studies (as it was with the liver and adipose tissue in the same model) [37,38].

## 4. Materials and Methods

### 4.1. Material

Fructose was purchased from Apipek (Bečej, Serbia). The anti-AMPKα 1/2 (sc-25792), drebrin (sc374269), anti-EGR1 (sc515830), anti-FosB (sc7230), anti-CaMKIIα (sc-9035), phospho-CaMKIIα (Thr286) (pCaMKIIα; sc-12886R), anti-GR (sc-8992), and anti-H6PDH (sc-67394) antibodies were from Santa Cruz Biotechnology (Dallas, TX, USA). The anti-GAD67 (MAB5406) and anti-PSD95 (MABN68) were from Merck (Darmstadt, Germany). The anti-parvalbumin antibody (P3088) was from Millipore-Sigma (Burlington, MA, USA). The phospho-AMPK (Thr172) (#41885), phospho-GR (Ser211) (#4161), and anti-FosB/delta FosB (#2251) antibodies were obtained from Cell Signalling Technology (Danvers, MA, USA). The anti-gephyrin (ab181382), anti-HSD1 (ab109554), anti-synaptophysin (ab14692), and anti-beta actin (ab8227) antibodies, as well as secondary anti-mouse (ab97046) and anti-rabbit (ab6721) horseradish peroxidase (HRP)-linked antibodies, were obtained from Abcam (Cambridge, UK).

### 4.2. Animals and Treatment

At the beginning of the 9-week treatment period, 2.5-month-old virgin female Wistar rats, bred in the animal facility of the Institute for Biological Research “Siniša Stanković”, were randomly divided into four experimental groups (*n* = 9 animals per group). The groups were as follows: a control group (C), fed with a standard diet (commercial food and drinking water); a fructose-fed group (F), supplied with the commercial food and 10% (*w*/*v*) fructose solution instead of drinking water; a stress-exposed group (S), supplied with the commercial food and drinking water and exposed to chronic unpredictable stressors (CUS); and a stress-exposed fructose-fed group (SF), supplied with the commercial food and 10% fructose solution and exposed to CUS.

The fructose concentration, as well as the type, sequence, and duration of the applied stressors, were chosen to resemble the modern human lifestyle [61,62]. The CUS protocol (modified from [61]) included following daily stressors: forced swimming in cold water for 10 min, physical restraint for 60 min, exposure to a cold room (4 °C) for 50 min, wet bedding for 4 h, switching cages for 2 h, rocking cages for 1 h, and a cage tilt (45°) overnight. The number (1 or 2) and type of daily stressor(s), as well as the onset of stress exposure (between 9 a.m. and 4 p.m. for all the stressors except for the overnight cage tilt) were randomly selected at the beginning of the experiment. A particular stressor was never applied on two consecutive days.

The animals were housed three per cage, kept under standard conditions at 22 °C with a 12 h light/dark cycle (lights on at 7 a.m.), and had constant veterinary care during the experiment. All the rats were fed ad libitum with commercial rat food [Laboratory Rat Food R20: 20% protein, 62.6% carbohydrate, and 3.2% fat, mineral and vitamin mix; Veterinary Institute Subotica, Serbia]. The food and fluid intakes were measured daily, while the body mass was recorded weekly. The food, fluid, and energy intake were expressed as the average weekly intake per body mass of the animals. The energy intake for the control and stressed rats was calculated by summing the energy ingested as food (food weight (g) x 11 kJ), while for the fructose-fed rats, it was calculated as the sum of the energy ingested by both food and the fructose solution (food weight (g) × 11 kJ + fructose intake (mL) × 1.72 kJ). All the animal procedures were in compliance with Directive 2010/63/EU on the protection of animals used for experimental and other scientific purposes, and were approved by the Ethical Committee for the Use of Laboratory Animals of the Institute for Biological Research “Siniša Stanković”, University of Belgrade (No. 02-11/14).

### 4.3. Behavioral Testing—Exploratory/Motor Activity in the Novel Open Arena

Female rats (6 per group, randomly chosen) were behaviorally examined during the 9th week of the exposure to fructose/stress, in the diestrus phase of the estrous cycle, which was determined by the analysis of vaginal smears (collected between 7:00 and 8:00 a.m.). If the animal met the condition of being in diestrus, it was transported to the testing room and was not subjected to the stressor planned for that day until testing was completed. The behavioral testing was performed during the period between 9:00 a.m. and 1:00 p.m.

The activity of the animals in an open field was used to assess their exploratory/motor and anxiety-like/thigmotactic behavior in response to an inescapable novel environment [63]. Following a habituation period (30 min in home cages) to the testing room, the rats were placed in Opto-Varimex cages (version 3.0A, Columbus Instruments, Columbus, OH, USA) and allowed to freely explore the novel environment for 30 min. Each cage (44.4 × 43.2 × 20 cm) was equipped with 15 infrared emitters located on the X and Y axes, with an equivalent number of receivers located on the opposite walls of the cage to track the full horizontal activity in a single cage. The additional Z axis sensors, placed above the animal, measured the vertical activity. The data were analyzed using the Auto-Track software (ATM3, version 4.51, Columbus Instruments). The Auto-Track interface collected data from the Opto-Varimex unit every 1/10th of a second and categorized the activity. The type of activity was determined by a user-defined box size (set to 4 beams). Locomotor activity was defined as a trespass of 4 consecutive photo-beams. The number of repetitive/stereotypy-like movements was defined as the number of repeated breaks of the same beam. Vertical activity was measured by recording the number of beams that were broken from the rearing of the animal. The Auto-Track interface has the ability to detect movements in 16 (4 × 4) equal fictional squares, allowing for the calculation of the number of entries and the time spent in the central zone (four squares in the middle).

After the termination of the 30 min exploratory period in the novel arena, the tested animals were returned to their home cages and the boxes were carefully cleaned and deodorized with a 20% ethanol cleaning solution to erase any smells that might interfere with the exploration by the next animal.

### 4.4. Tissue Preparation

The animals (6 per group) were killed in the diestrus phase by rapid decapitation, at least 24 h after the application of the last stressor. This period included overnight fasting, during which all the experimental groups were provided only with drinking water. After decapitation, the medial prefrontal cortex was isolated on ice; the tissue was frozen in liquid nitrogen and kept at −70 °C until use. For the preparation of whole cell extracts, the tissue was homogenized in ice-cold RIPA buffer (50 mM Tris-HCl, pH of 7.4, containing 150 mM NaCl, 10 mM EDTA, 10 mM EGTA, 1% NP40, 0.1% SDS, 2 mM dithiothreitol, and protease and phosphatase inhibitors) with 20 strokes of a glass homogenizer. The homogenates were sonicated for 3 × 5 s at 1 A and 50/60 Hz on ice, incubated on ice for 30 min with continuous agitation and frequent vortexing, and centrifuged for 20 min at 14,000× *g* and 4 °C. The obtained supernatants were stored at −70 °C.

### 4.5. SDS Polyacrylamide Gel Electrophoresis and Western Blotting

After boiling in Laemmli’s sample buffer, 40 µg of proteins was resolved on an SDS polyacrylamide gel. The Western transfer of the proteins from the gels to the PVDF membranes was performed in 25 mM Tris buffer with a pH of 8.3 containing 192 mM glycine and 20% (*v*/*v*) methanol, at 135 mA overnight in a Mini Trans Blot Electrophoretic Transfer Cell (Bio Rad Laboratories, Hercules, CA, USA). The membranes were blocked by PBS (1.5 mM KH_2_PO_4_, 6.5 mM Na_2_HPO_4_, 2.7 mM KCl, 0.14 M NaCl, pH of 7.2) containing 5% BSA. After blocking, the membranes were incubated with the respective primary antibody, followed by HRP-conjugated secondary antibodies. A protein load correction in all the samples was achieved by probing the membranes for β-actin. Immuno-positive bands were visualized using the enhanced chemiluminiscent (ECL) method, and were quantified by the iBright FL1500 Imaging System Software (version 4.0.1., Thermo Fisher Scientific, Waltham, MA, USA).

### 4.6. Determination of Plasma Corticosterone

For plasma preparation, trunk blood was collected at the end of the experiment in EDTA-coated tubes, centrifuged at 3000 rpm for 10 min, and stored at −20 °C until use. The corticosterone (CORT) concentration in the plasma was determined using the Corticosterone EIA kit according to the manufacturer’s instructions (Immunodiagnostic Systems Ltd., Boldons, UK).

To assess the corticosterone level before and immediately after the application of stressors, blood was collected via a tail snip at experimental weeks 1 and 8. This procedure was used to assess whether and to which stressors the exposed experimental animals became accustomed.

### 4.7. Statistical Analysis

The data are given graphically, as the mean ± SD, with individual data plots along the column bars. The statistical analysis was performed using parametric or non-parametric tests (STATISTICA 8.0. Software, StatSoft, Inc., Tulsa, OK, USA), depending on the distribution of the data sets of interest. The difference was considered significant at *p* < 0.05.

The influence of liquid fructose supplementation, stress, and their interaction on behavior was assessed using the Kruskal–Wallis *H* test and between-group comparisons were performed using the Mann–Whitney *U*-test. The within-session habituation was assessed using the Wilcoxon test, comparing the activity achieved during the first and last five minutes of a total of 30 min of testing.

The influence of fructose, stress, and their interaction on the expression of proteins of interest was assessed either using the Kruskal–Wallis *H* test, with between-group comparisons performed using the Mann–Whitney *U*-test, or using a one-way ANOVA followed by Tukey’s post hoc test, depending on the data distribution.

To assess the effects of stress on feeding behavior, the Student’s *t*-test was used for comparisons between C and S and between the F and SF groups.

## 5. Conclusions

In conclusion, long-term fructose consumption alone is not related to affected behavioral control in response to an environmental novelty, at least in female rats. Fructose in combination with CUS prevents some of its neurobehavioral effects, but also contributes new ones not evident with either treatment alone. Thus, CUS should be viewed as an additional variable that contributes to higher fructose consumption and markedly sculpts the outcomes of its long-term intake.

## Figures and Tables

**Table 1:** Summary of the results for behavioral outcomes and biochemical alterations in the medial prefrontal cortex and plasma due to 9 weeks of fructose feeding (F), chronic unpredictable stress exposure (S), or both (SF) in female Wistar rats. The meaning of the symbols is as follows: no change compared to the control group (/), an increase compared to the control group (↑), and a decrease compared to the control group (↓).

Parameter	Experimental Groups		
	F	S	SF
**Novelty-induced behavioral response**			
Locomotor activity	/	↑	/
Stereotypy-like activity	/	/	/
Vertical/rearing activity	/	↑	/
Time spent in the central zone	/	/	↓
**Protein indicators of neuronal activation**			
EGR1	/	/	/
FosB	/	↓	/
ΔFosB	/	/	/
**Protein indicators of synaptic potentiation**			
CaMKIIα	↓	↓	/
pCaMKII^Thr286^	↑	↑	/
pCaMKII^Thr286^/CaMKIIα	↑	↑	/
**Protein indicators of altered synaptic plasticity**			
PSD95	/	/	/
Synaptophysin	/	/	/
Gephyrin	/	/	↓
Drebrin	/	/	↑
**Mediators of the stress response**			
Glucocorticoid receptor	/	↓	↓
Corticosterone	/	/	/
**Markers of the GABAergic system**			
Parvalbumin	/	↑	/
GAD67	/	/	/
**Protein sensors of cellular energy status**			
AMPK	/	/	/
pAMPK^Thr172^	/	/	/
pAMPK^Thr172^/AMPK	/	/	/

## Data Availability

The original contributions presented in this study are included in the article/Supplementary Materials; further inquiries can be directed to the corresponding author.

## Supplementary Materials

The following supporting information can be downloaded at: https://www.mdpi.com/article/10.3390/ijms252111721/s1.
