# Supplementary material for: High-Fructose Diet and Chronic Unpredictable Stress Modify Each Other’s Neurobehavioral Effects in Female Rats
Source: Int J Mol Sci. 2024 Oct 31;25(21):11721. doi: 10.3390/ijms252111721 (PMC11546065; doi:10.3390/ijms252111721)
Supplement: Supplementary file 1 [file ijms-25-11721-s001.zip › ijms-3259606-supplementary.pdf]

**ECL iBright Western Blot Imaging Systems | Thermo Fisher**<br>C – control<br>F – fructose<br>S – stress<br>SF – stress+fructose	
EGR1	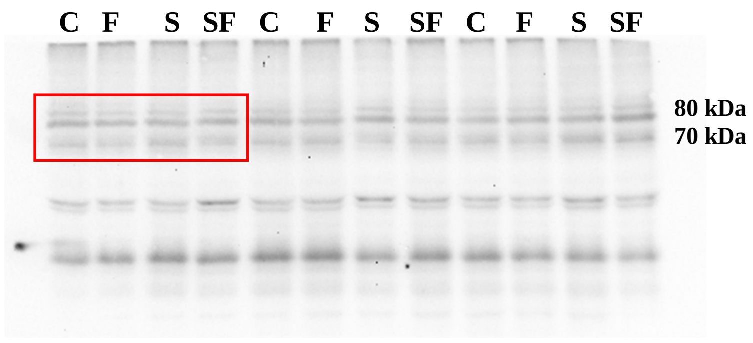
β-Actin (EGR1)	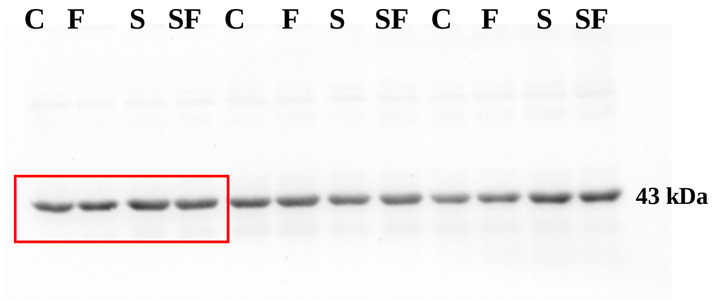
FosB	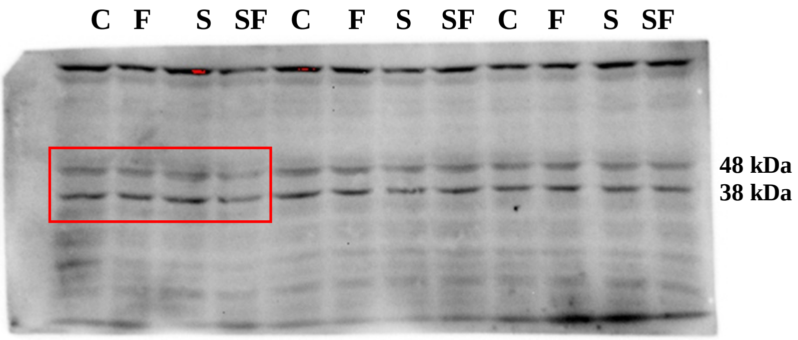
β-Actin (FosB)	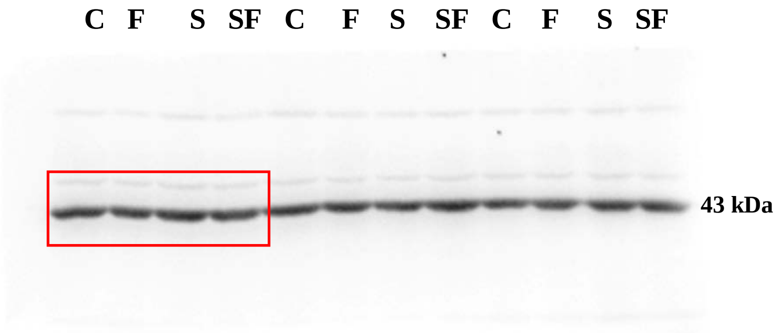

ΔFosB	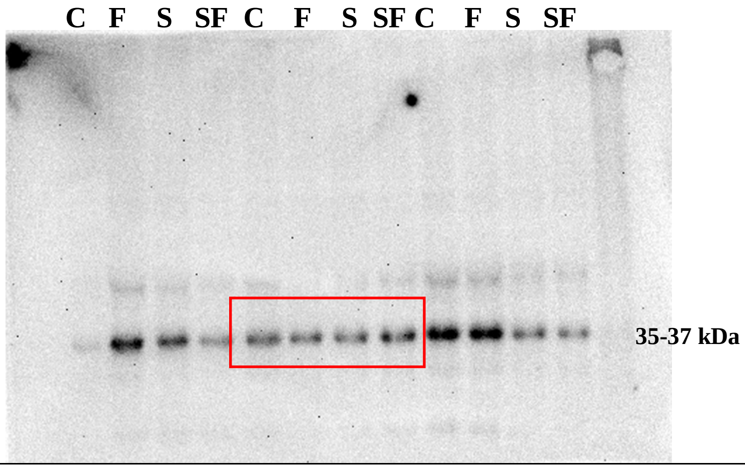
β-Actin (FosB)	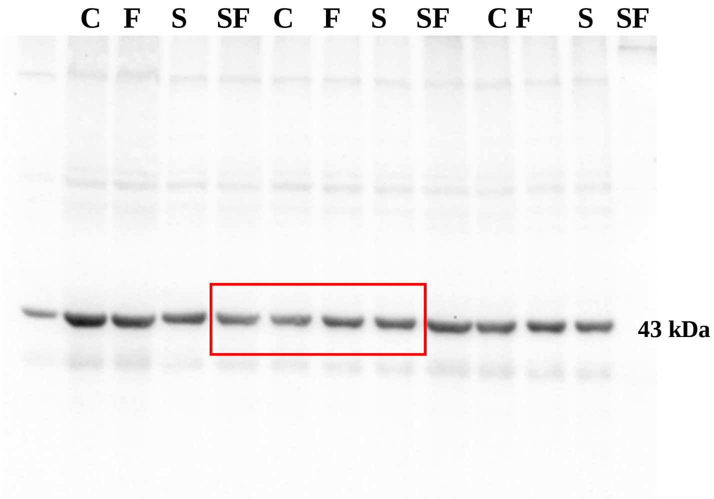
pCaMKIIThr286	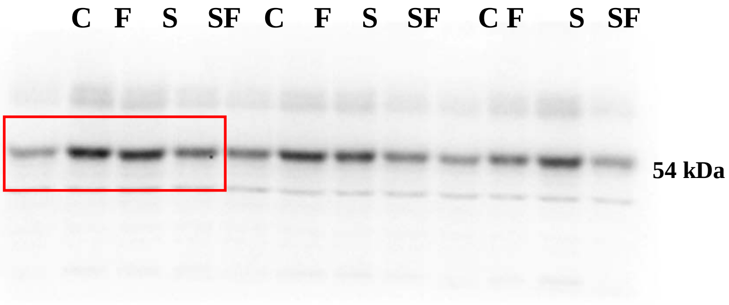
CaMKII	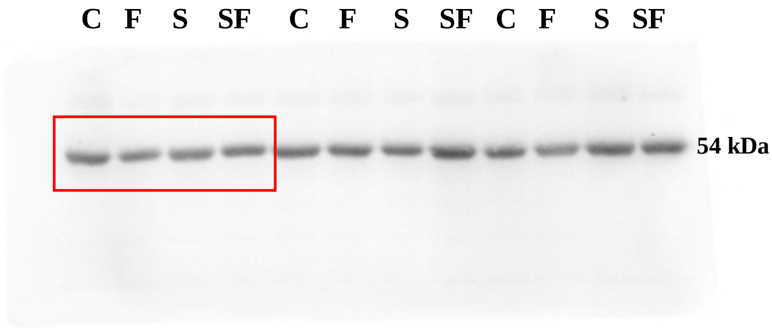

β-Actin (CaMKII)

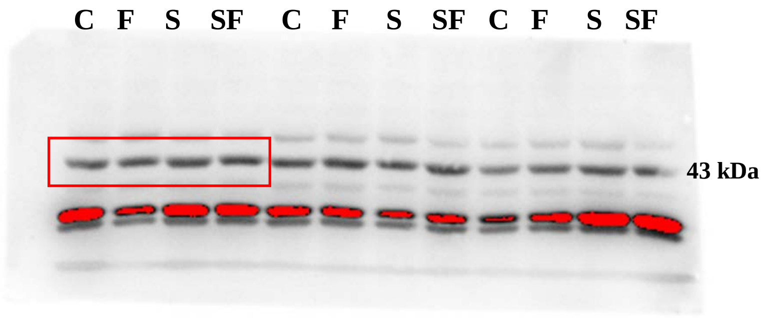

PSD95

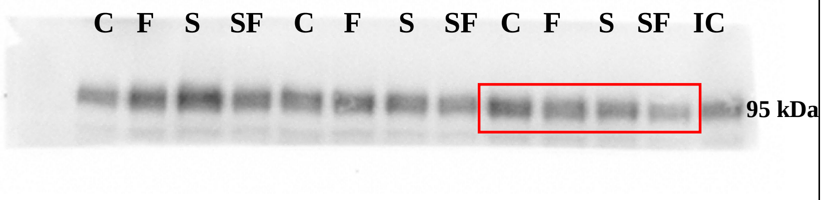

β-Actin (PSD95)

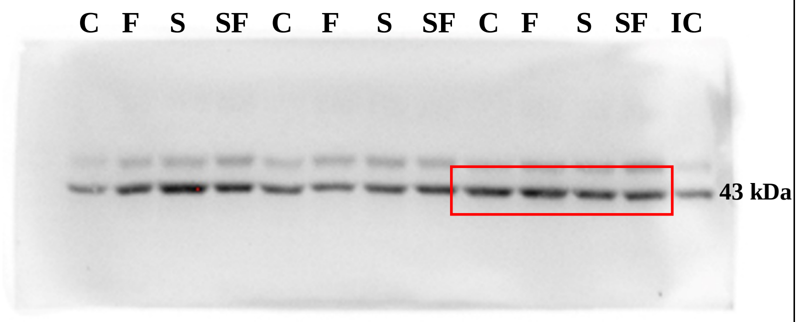

Synaptophysin

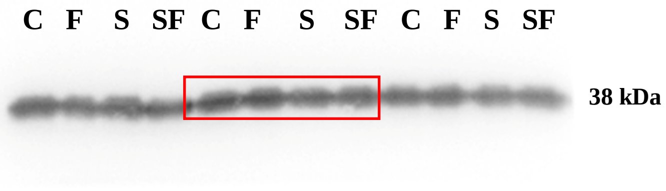

β-Actin (Synaptophysin)

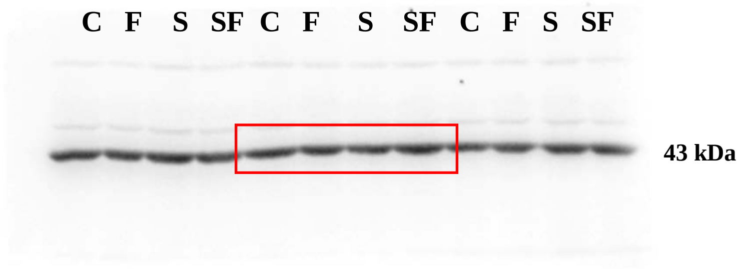

Gephryn	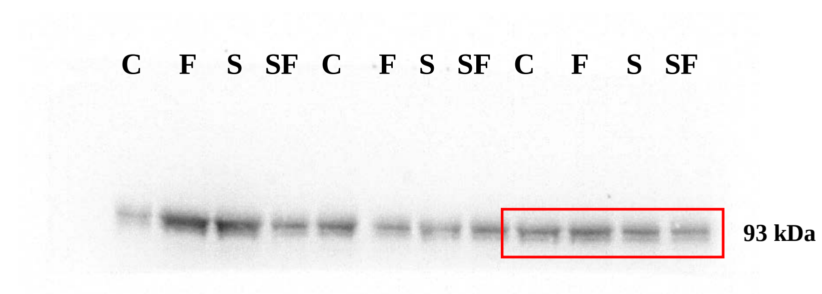
β-Actin (Gephryn)	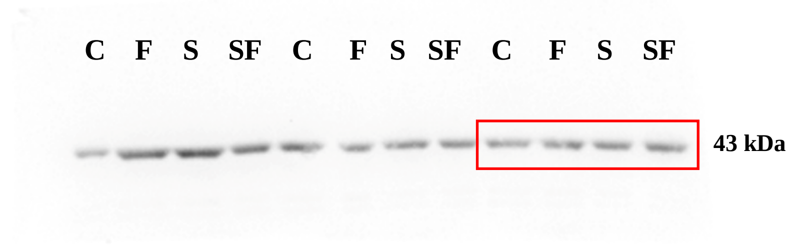
Drebrin	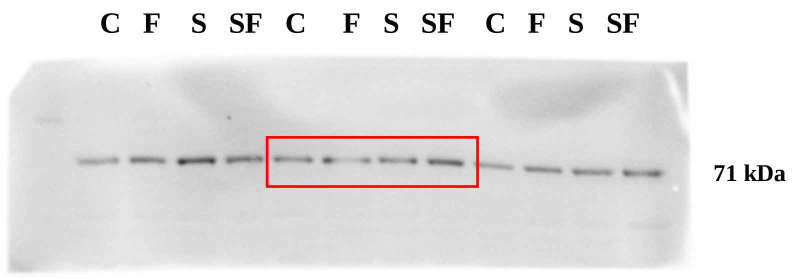
β-Actin (Drebrin)	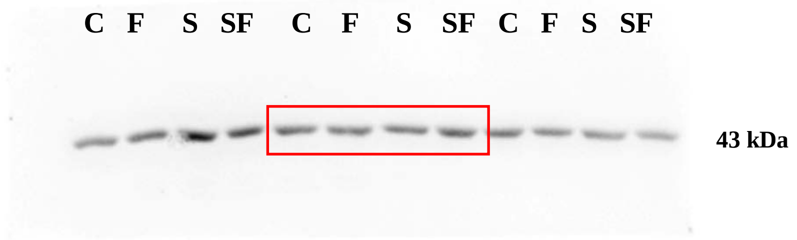
GR	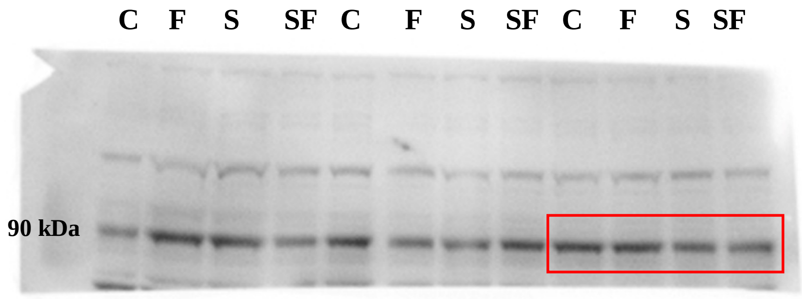

β-Actin (GR)	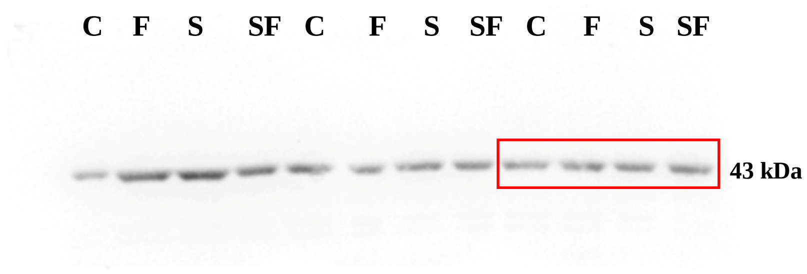
Parvalbumin	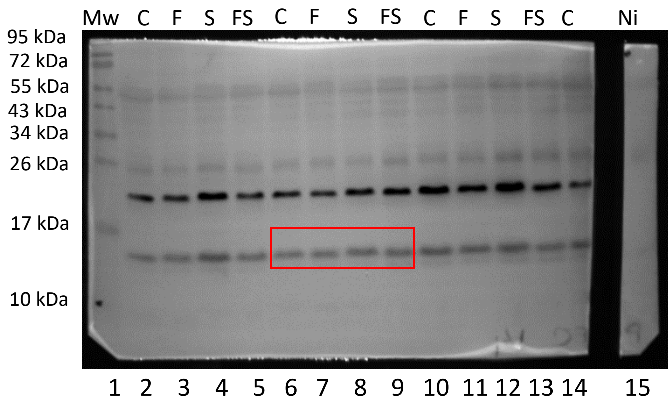
β-Actin (Parvalbumin)	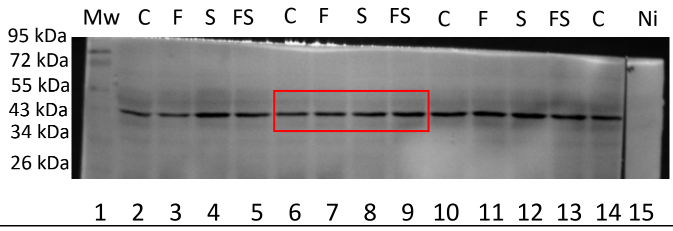
GAD67	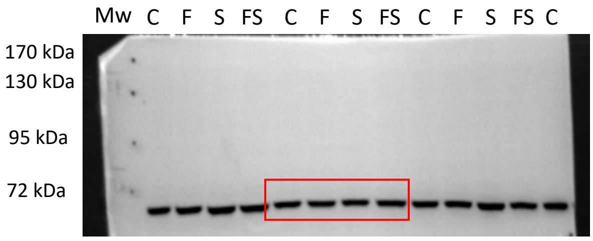
β-Actin (GAD67)	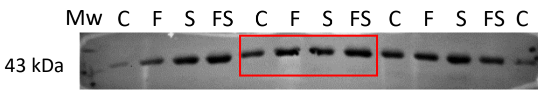

<table>
<tr><td>pAMPKThr172</td><td>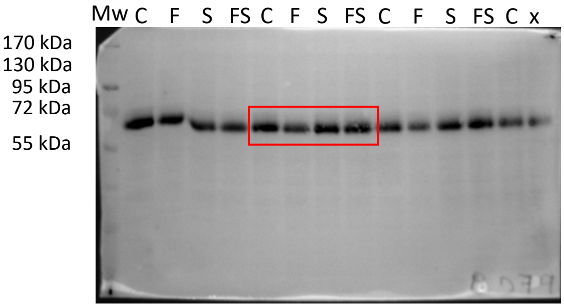
</td></tr>
<tr><td>β-Actin (pAMPKThr172)</td><td>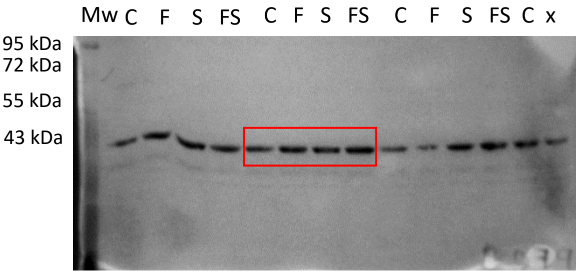
</td></tr>
<tr><td>AMPK</td><td>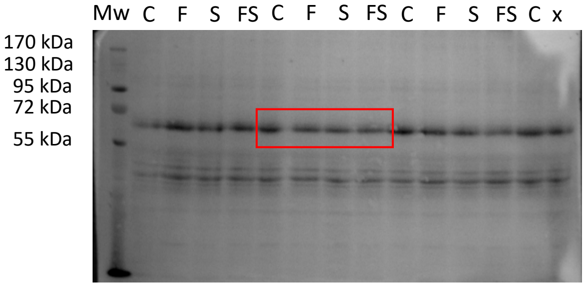
</td></tr>
<tr><td>β-Actin (AMPK)</td><td>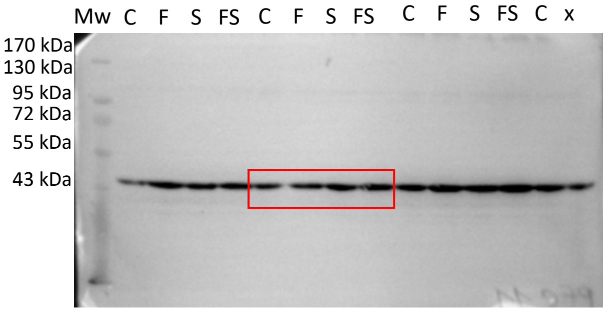
</td></tr>
</table>
